# Soluble programmed cell death ligand-1 as a predictive biomarker for severity and poor prognosis in pulmonary tuberculosis

**DOI:** 10.1080/07853890.2025.2527364

**Published:** 2025-07-09

**Authors:** Xiaojue Wang, Weibing Lin, Huimin Li, Sibo Long, Jun Yan, Yiheng Shi, Hongtao Zhang, Xinting Yang, Ling Yi, Guirong Wang

**Affiliations:** a Department of Cancer Research Center, Beijing Chest Hospital, Capital Medical University, Beijing Tuberculosis and Thoracic Tumor Research Institute, Beijing, China; b Department of Clinical Laboratory, Beijing Chest Hospital, Capital Medical University, Beijing Tuberculosis and Thoracic Tumor Research Institute, Beijing, China; c Tuberculosis Department, Beijing Chest Hospital, Capital Medical University, Beijing, China

**Keywords:** Pulmonary tuberculosis, sPD-L1, severity, predictive marker, prognosis**Introduction**

## Abstract

**Background:**

We aimed to assess whether soluble programmed death-ligand 1 (sPD-L1) could serve as a new biomarker for PTB.

**Methods:**

Plasma sPD-L1 levels in the discovery cohort were analyzed through flow cytometry and validated by sandwich ELISA. Pleural effusion sPD-L1 levels were measured using ELISA.

**Results:**

In the discovery cohort, sPD-L1 levels in the severe (SE, *n* = 44), non-severe (non-SE, *n* = 34) and HC (*n* = 10) group were 67.41 (30.14–126.41), 26.75 (11.00–52.35) and 14.6 (10.78–21.91) pg/ml, respectively. The sPD-L1 levels in SE patients were significantly higher than those in both non-SE patients and HCs (*p* < 0.0001). These findings were confirmed in the validation cohort with sPD-L1 levels significantly higher in SE (*n* = 60,763.81 pg/ml) compared to both non-SE patients (*n* = 80, 318.30 pg/ml) and HCs (*n* = 79, 202.33 pg/ml)(*p* < 0.0001). Receiver operating characteristic (ROC) analysis demonstrated plasma sPD-L1 could distinguish SE from non-SE PTB with an AUC of 0.8058 (95% CI 0.7308–0.8808). sPD-L1 levels showed positive correlations with inflammatory markers, such as neutrophil percentage (NEU%, *r* = 0.5743, *p* < 0.0001), neutrophil-to-lymphocyte ratio (NLR, *r* = 0.5952, *p* < 0.0001). Survival analysis revealed shorter survival times in groups with higher sPD-L1 (≥445.1 pg/ml, *p* = 0.0006). In addition, sPD-L1 levels in tuberculous pleural effusion (TPE) were significantly higher than malignant pleural effusion (MPE) (1964.72 versus 159.38 pg/ml, *p* < 0.001), showing diagnostic performance (AUC = 0.9837) similar to adenosine deaminase (AUC= 0.9859).

**Conclusion:**

Elevated plasma sPD-L1 may be a predictive marker for both disease severity and poor prognosis in PTB patients. Pleural effusion sPD-L1 levels might potentially function as an adjunctive marker for differentiating TPE from MPE.

## Introduction

Programmed cell death ligand 1 (PD-L1), which is the ligand of coinhibitory immune checkpoint protein-programmed death protein 1 (PD-1), is expressed on various human cells, such as T or B cells, macrophages and dendritic cells, etc. [1–3]. The binding of PD-1 and PD-L1 leads to the suppression of T cell activity and T cell mediated cytotoxicity, resulting in driving the plethora inflammation to a reasonable range, keeping the immune homeostasis, maintaining self-tolerance and protecting tissues from damage caused by the immune system [1,4]; or dampening of the anti-cancer immune response and facilitating immune escape of cancer cells [1,2,5,6]. Several inhibitors against PD-1/PD-L1 pathway are approved in non-small cell lung cancer and many more are under clinical development [2,4,6].

PD-L1 also exists in soluble isoforms and two potential mechanisms for their occurrence were described: splice variants and shedded proteins [2,7,8]. In melanoma and non-small cell lung cancer patient, several variants have been described that are induced by specific cytokines profiles (IFN-γ, IFN-α, and TNF-α) [2,8]. sPD-L1 can be measured in peripheral blood of healthy individuals and patients with various conditions including cancer and inflammatory diseases. In cancer patients, sPD-L1 has been investigated as a possible predictive and prognostic biomarker for immunotherapy [2,3,9–11]. In inflammation status, such as sepsis, acute pancreatitis, and acute coronary syndrome, plasma or serum sPD-L1 may reflect disease severity, immune dysfunction and poor clinical outcomes [12–14].

Some studies have found that circulating sPD-L1 levels were elevated in tuberculosis (TB) patients compared to healthy controls, with particularly high concentration observed in tuberculous pleural effusion (TPE) [15,16]. However, no studies to date have established an association between sPD-L1 levels and disease severity or prognosis in PTB. In this study, we detected the sPD-L1 levels in both plasma and TPE from TB patients and found that elevated sPD-L1 levels could predict disease severity and poor prognosis, and distinguish TPE from malignant pleural effusions (MPE).

## Methods

### Patients and healthy controls

This study included three cohorts. The first one is plasma sPD-L1 discovery cohort, including 78 pulmonary tuberculosis (PTB) patients and 10 healthy controls (HCs); the second is plasma sPD-L1 verification cohort, including 140 PTB patients and 79 HCs; the third is the pleural effusion cohort, including 85 TB patients with TPE, and 40 untreated lung cancer patients with MPE. These patients were diagnosed with PTB through Xpert MTB/RIF and/or sputum culture at Beijing Chest Hospital from May 2022 to November 2022. The classification criteria for severe (SE) and non-severe (non-SE) PTB patients were based on a combination of radiographic and clinical criteria [17]. SE PTB defined as the presence of bilateral cavitary disease or extensive parenchymal damage on chest radiography (CXR), and/or long-term bacterial discharge, PO2 < 80 mmHg or oxygenation index <300 mmHg. Non-SE PTB defined as intrathoracic lymph node TB without airway obstruction; uncomplicated TB pleural effusion; or paucibacillary, non-cavitary disease confined to one lobe of the lungs and without a miliary pattern. Exclusion criteria were as follows: (1) current use of immunosuppressive drugs or corticosteroids, (2) viral hepatitis, (3) acute infection, (4) incomplete clinical data, (5) autoimmunity disease, (6) second malignancy within the last 10 years.

This study was approved by the Ethics Committee of Beijing Chest Hospital (approval number: YNLX-2022-006), and written informed consent were obtained from all participants.

### Sample collection and processing

Peripheral blood was collected in EDTA anticoagulant tubes within 7 days of admission. After centrifugation (3,000 rpm, 10 min, 4 °C), plasma was aliquoted and stored at −80 °C within 4 h after collection. For pleural effusion, 10 ml of pleural fluid was obtained by thoracentesis and aliquoted and stored at −80 °C for subsequent analysis.

### sPD-L1 quantification by flow cytometric

First, color-coded magnetic beads conjugated with anti-PD-L1 capture antibodies were incubated with plasma (25 µL, 1:2 dilution) or reference standards in 96-well plates and incubated for 2 h on a shaker. Later, biotin-labeled quantified antibody was added and incubated for 1 h at room temperature. Then, PE-labeled streptavidin (SA-PE) was added, and incubated for 30 min at room temperature. The sPD-L1 concentrations were analyzed using an LSRFortessa flow cytometer.

### Sandwich ELISA and blood sPD-L1 detection

A previously established ELISA protocol [12] was utilized to measure sPD-L1 levels in plasma and pleural effusion samples. Briefly, anti-human PD-L1 mAbs were generated by Hybridoma prepared in our lab and purified by affinity chromatography. The two mAbs used in ELISA were carefully characterized and obtained patent authorization. ELISA plates were coated with capture mAb at 2 μg/mL, blocked with 5% skim milk (BD Difco, Sparks, MD, USA), then diluted (1:25) plasma or pleural fluid samples were added to the plates and incubated for 2 h at room temperature (RT). Subsequently, biotinylated anti-PD-L1 monoclonal antibody (2 μg/mL) and streptavidin–HRP (1:3000, Cell Signaling Technology) was added. The positive reaction was developed using the TMB substrate reagent set (BD OptEIA, San Diego, CA, USA). All samples were tested in duplicate. Recombinant human PD-L1 (Sino Biological, Inc.) was used as a standard.

### Statistical analysis

Statistical analyses were performed using SPSS version 24.0 and GraphPad Prism 10. Normally distributed continuous variables were shown as mean ± standard deviation (SD) and compared using the independent sample *t* test. Non-normally distributed data was presented as median (interquartile range, IQR) and analyzed using the Mann–Whitney *U* test. One-way ANOVA was used for multiple group comparisons, followed by post hoc pairwise comparisons. Spearman’s rank correlation coefficient was calculated to assess correlations between sPD-L1 levels and laboratory parameters. Patient survival was analyzed using the log-rank test. A *p* value of less than 0.05 was considered statistically significant.

## Results

### Pantients’ clinical characteristics

We mainly showed the clinical characteristics of patients from validation cohort, which included 60 SE and 80 non-SE patients. The median ages were comparable between SE and non-SE patients (56 years,36–69 versus 58 years, 39–69; *p* = 0.674). The proportions of patients with fever (43.33 versus 21.25%, *p* = 0.006) and wheezing (23.33 versus 6.25%, *p* = 0.007) were significantly higher in the SE group than in the non-SE group (Supplementary Table 1). The smear positivity rate of *Mycobacterium tuberculosis* (Mtb) was also higher in the SE group than in the non-SE group (60.00 versus 42.50%, *p* = 0.018).

Regarding laboratory data, the white blood cell count (WBC, 8.25 versus 6.50 × 10^9^/L; *p* = 0.009), neutrophil percentage (NEUT%, 83.40 versus 67.20%; *p* < 0.001), neutrophil-to-lymphocyte ratio (NLR, 9.91 versus 3.28; *p* < 0.001), high-sensitivity C-reactive protein (hs-CRP, 56.03 versus 13.97 mg/L; *p* = 0.003), lactate dehydrogenase (LDH, 189.00 versus 175.00 U/L; *p* = 0.005), d-dimer (d–d, 2.77 versus 0.86 mg/L; *p* = 0.002), and adenosine deaminase (ADA, 17.50 versus 11.85 U/L; *p* < 0.001) were significantly higher in SE than in non-SE patients. Meanwhile, lymphocyte percentage (LY%, 8.45 versus 20.40%; *p* < 0.001), total protein (TP, 60.05 versus 69.30 g/L; *p* < 0.001) and albumin (Alb, 30.30 versus 37.80 g/L; *p* < 0.001) were significantly lower in SE than in non-SE patients. All these data were summarized in Table 1 (left panel).

**Table 1.** Comparison of laboratory data between SE and non-SE patients.

Inspection items	Plasma cohort			Multivariable	
	Non-SE (*n* = 80)	SE (*n* = 60)	*P* value	OR (95% Cl)	*P* value
WBC (×10^9^/L)	6.50 (5.01–8.91)	8.25 (5.70–10.93)	0.009	1.189 (0.983–1.438)	
PLT (×10^9^/L)	274.00 (208.25–323.00)	265.50 (206.00–381.00)	0.494		
LY%	20.40 (12.35–32.33)	8.45 (4.08–12.08)	0.001	0.993 (0.850–1.160)	
NEUT%	67.20 (55.60–78.28)	83.40 (77.13–90.80)	0.001	0.991 (0.868–1.131)	
NLR	3.28 (1.74–6.35)	9.91 (6.33–21.53)	0.001	0.812 (0.685–0.963)	0.016
TP (g/L)	69.30 ± 9.43	60.05 ± 13.78	0.001	1.140 (1.046–1.243)	0.003
TBIL (µmol/L)	10.60 (8.33–13.85)	11.35 (8.23–17.43)	0.146		
ADA (U/L)	11.85 (8.33–16.68)	17.50 (11.88–21.40)	0.001	0.925 (0.852–1.005)	
LDH (U/L)	175.00 (147.50–200.75)	189.00 (148.25–268.00)	0.005	1.001 (0.993–1.009)	
PCT (ng/ml)	0.03 (0.02–0.07)	0.15 (0.06–0.29)	0.695		
d–d (mg/L)	0.86 (0.40–2.52)	2.77 (1.09–5.05)	0.002	0.898 (0.707–1.140)	
Hs-CRP (mg/L)	13.97 (2.88–54.01)	56.03 (34.32–76.84)	0.003		0.030
Alb (g/L)	37.80 (32.60–40.98)	30.30 (25.83–33.88)	0.001	1.024 (0.920–1.140)	
sPD-L1 (pg/ml)	318.30 (227.08–443.90)	763.81 (472.90–852.77)	0.001	0.997 (0.996–0.999)	0.007

WBC white blood cell count, PLT platelet count, LY% percentage of lymphocytes, NEUT% percentage of neutrophils, NLR neutrophil-to-lymphocyte ratio, TP total protein, TBIL total bilirubin, ADA adenosine deaminase, LDH lactic dehydrogenase, PCT procalcitonin, d–d d–Dimer, hs-CRP C-reactive protein, Alb albumin.

Data were presented as mean ± standard deviation (SD) or median (interquartile range); variables with significant differences were included in the multivariate analysis.

### Elevated plasma sPD-L1 is a predictive biomarker of disease severity

**Figure 1.**
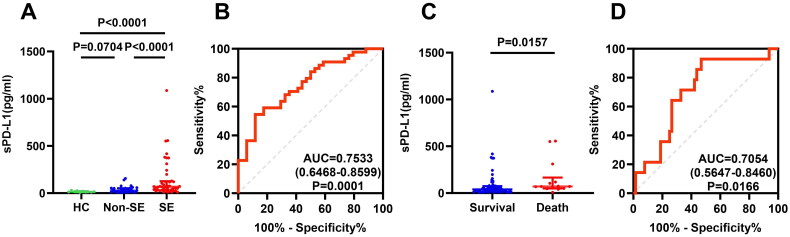
Comparison of the plasma sPD-L1 level between pulmonary tuberculosis (PTB) patients and healthy controls(HCs) through immunofluorescence assay. (A) Comparison of plasma sPD-L1 levels among HCs (*n* = 10), non-severe (non-SE, *n* = 34) and severe (SE) PTB patients (*n* = 44). Plasma sPD-L1 levels were measured using an immunofluorescence assay, the data are presented as median ± interquartile ranges (IQRs), and One-way ANOVA with Dunn’s post hoc test was used to get the *P* value. (B) Receiver-operating characteristic (ROC) curve analysis was performed to evaluate the sensitivity, specificity of plasma sPD-L1 levels in distinguishing PTB patients from HCs (area under the curve, AUC = 0.7533, *P* = 0.0001). (C) Comparison of plasma sPD-L1 levels in PTB patients who survived (*n* = 64) and those who died (*n* = 14). The Mann–Whitney test was used (*P* = 0.0157). (D) ROC curve analysis of sPD-L1 levels was conducted to assess its potential predicting value in PTB patients’ prognosis, with an AUC of 0.7054 (*P* = 0.0166).

The plasma sPD-L1 levels in discovery cohort were detected using immunofluorescence assay. The sPD-L1 levels in the SE (*n* = 44), non-SE group (*n* = 34) and HC (*n* = 10) group were 67.41 (30.14–126.41), 26.75 (11.00–52.35) and 14.6 (10.78–21.91) pg/ml, respectively. The sPD-L1 levels in SE patients were significantly higher than those in non-SE patients and HCs (Figure 1A, *p* < 0.0001),but there was no significant difference between non-SE patients and HCs (Figure 1A, *p* = 0.0704). ROC curve analysis indicated that plasma sPD-L1 could serve as a biomarker for distinguishing the severity of TB, with the area under the curve (AUC) of 0.7533 (95% CI 0.6468–0.8599, Figure 1B). Furthermore, the plasma sPD-L1 levels in dead group (*n* = 14) were significantly higher than those in survival group (*n* = 64) (70.64, 49.29–166.21 versus 38.90, 17.46–73.80 pg/ml; *p* = 0.0157, Figure 1C). sPD-L1 can be used as an indicator of PTB patients’ poor prognosis, with an AUC of 0.7054 (95% CI: 0.5647–0.8460; Figure 1D).

**Figure 2.**
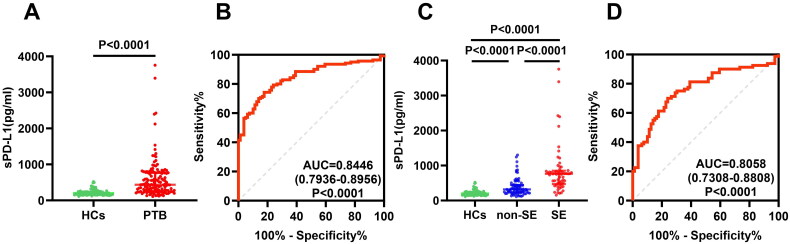
Comparison of the plasma sPD-L1 level between PTB patients and HCs through sandwich ELISA. (A) Comparison of sPD-L1 level in PTB patients (*n* = 140) and HCs (*n* = 79). All samples were assayed in duplicate, and one representative result from two experiments was shown. The Mann–Whitney test was used to get the *p* value (*P* < 0.0001). The data are presented as the medians ± IQRs. (B) ROC curve was used to assess the ability of sPD-L1 to differentiate TB patients from HCs. (C) Comparison of sPD-L1 levels in HCs (*n* = 79), non-SE (*n* = 80), and SE (*n* = 60) patients. One-way ANOVA with Dunn’s post hoc test was used. The result showed that sPD-L1 levels in SE patients were significantly higher than non-SE and HCs (*P* < 0.0001). There was also significant difference between non-SE and HCs (*P* < 0.0001). (D) The ROC curve was used to evaluate the value of sPD-L1 levels to distinguish SE patients from non-SE patients.

To further validate plasma sPD-L1 levels, we measured sPD-L1 levels in another cohort through ELISA. The plasma sPD-L1 levels in PTB patients were significantly elevated compared to HCs (434.59, 256.32–769.73 versus 202.33, 157.71–242.65 pg/ml; *p* < 0.0001, Figure 2A). ROC curve analysis indicated that plasma sPD-L1 level distinguished PTB patients from HCs, with an AUC of 0.8446 (95% CI 0.7936–0.8956; Figure 2B). The SE group (*n* = 60) had significantly higher sPD-L1 levels than the non-SE group (*n* = 80) (Figure 2C, *p* < 0.0001) and sPD-L1 can indicate the severity of PTB patients, with an AUC of 0.8058 (95% CI 0.7308–0.8808; Figure 2D). Multivariable analysis further confirmed sPD-L1 as an independent predictor of severe PTB (Table 1, right panel).

### The sPD-L1 level can distinguish tuberculous pleural effusion from malignant pleural effusion

**Figure 3.**
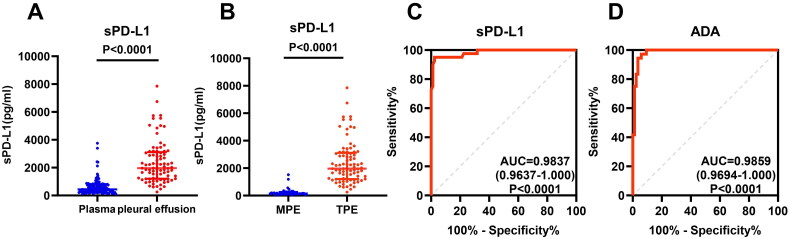
Comparison of the sPD-L1 concentration in pleural fluid from different types of patients. (A) Comparison of sPD-L1 levels in PTB patients’ blood and pleural effusion. The sPD-L1 levels in PTB patients’ pleural effusion (*n* = 85) were further increased than those in their blood (*n* = 140, Mann–Whitney test, *P* < 0.0001). The data are presented as median ± IQRs. (B) Comparison of sPD-L1 levels in tuberculous pleural effusion (TPE) and malignant pleural effusion (MPE). The sPD-L1 levels in TPE (*n* = 85) were significantly higher than those in MPE (*n* = 40, Mann–Whitney test, *P* < 0.0001). (C) The ROC curve of sPD-L1, AUC = 0.9837. (D) The ROC curve of adenosine deaminase (ADA) from the same PTB patient group, AUC = 0.9859. All samples were assayed in duplicate, and one representative result from two experiments.

In addition to plasma measurement, we also quantified the sPD-L1 levels in TPE (*n* = 85) and MPE (*n* = 40) from lung cancer patients. The sPD-L1 levels in pleural fluid was significantly higher than plasma in TB patients (1964.72, 1202.55–3115.54 versus 434.59, 256.32–769.73 pg/ml; *p* < 0.0001, Figure 3A). Moreover, by comparing with MPE, sPD-L1 in TPE were significantly higher (1964.72, 1202.55–3115.54 versus 159.38, 106.08–262.12 pg/ml; *p* < 0.0001, Figure 3B). ROC analysis revealed sPD-L1 level could be used to distinguish TPE from MPE (AUC = 0.9837, 0.9637–1.000; *p* < 0.0001, Figure 3C), with performance comparable to ADA (AUC = 0.9859, 0.9694–1.000; *p* < 0.0001, Figure 3D).

### High plasma sPD-L1 levels reflect high inflammatory state of PTB

**Figure 4.**
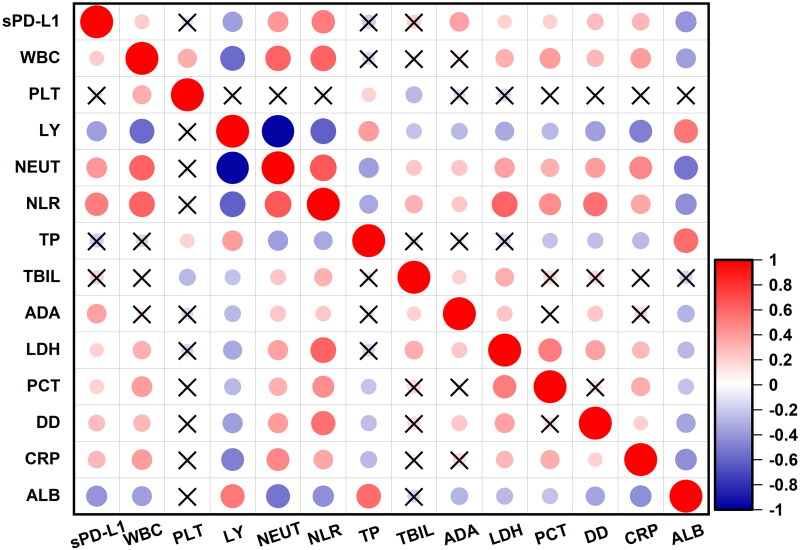
Correlations between sPD-L1 level and laboratory examination index in PTB patients. Heatmap showed the correlation coefficients between PTB patients’ sPD-L1 levels and their various laboratory indices (*n* = 140), including WBC (white blood cell count), PLT (platelet count), LY (percentage of lymphocyte), NEUT (percentage of neutrophils), NLR (neutrophil-to-lymphocyte ratio), TP (total protein), TBIL (total bilirubin), ADA (adenosine deaminase), LDH (lactic dehydrogenase), PCT (procalcitonin), d-Dimer, hs-CRP( high-sensitivity C-reactive protein), and ALB ( albumin). Positive correlations are represented in red, while negative correlations are shown in blue, no correlations are marked with a black cross. The size of the circles indicates the strength of the correlation, with larger circles representing stronger correlations. Spearman’s rank correlation coefficient was used to assess correlations between sPD-L1 levels and laboratory parameters.

At the same time, the correlations between sPD-L1 levels and laboratory parameters were analyzed. There were positive correlations between sPD-L1 levels and various inflammatory markers, including WBC (*r* = 0.3136, *p* = 0.0002), NEU% (*r* = 0.5743, *p* < 0.0001), NLR (*r* = 0.5952, *p* < 0.0001), hs-CRP (*r* = 0.5885, *p* < 0.0001), ADA (*r* = 0.5220, *p* < 0.0001), LDH (*r* = 0.2451, *p* = 0.0035), PCT (*r* = 0.6622, *p* < 0.0001), and d–d (*r* = 0.5782, *p* < 0.0001). Meanwhile, sPD-L1 levels showed negative correlations with LY% (*r* = –0.5993, *p* < 0.0001), TP (*r* = –0.3103, *p* = 0.0002), and Alb (*r* = –0.5260, *p* < 0.0001). There was no significant correlation between sPD-L1 levels and platelet count (PLT) (Figure 4).

### High plasma sPD-L1 levels indicate poor prognosis in PTB patients

**Figure 5.**
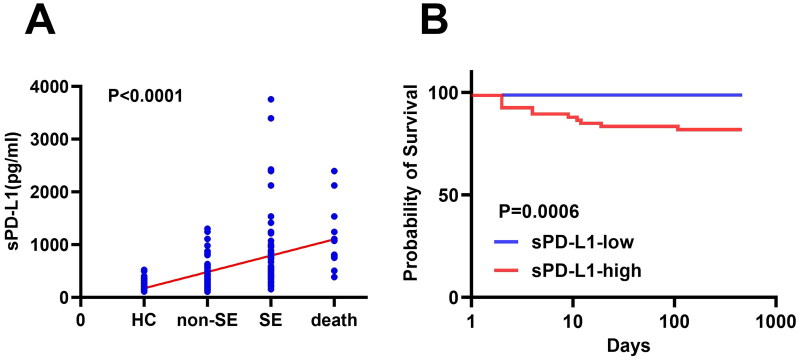
Relationships between the sPD-L1 level and patient prognosis. (A) Plasma sPD-L1 levels among the HC (*n* = 79), non-severe (*n* = 80), SE (*n* = 60), and death (*n* = 13) groups were gradually increased. As of August 30, 2023, 13 patients died (12 SE and 1 non-SE), trend test analysis was used (*P* < 0.0001). (B) PTB patients with higher sPD-L1 levels had a shorter survival time. PTB patients (*n* = 140) were subdivided into two groups according to the cutoff value (445.1pg/ml), patients with lower (*n* = 74) and higher (*n* = 66) sPD-L1 levels, had a significant difference in survival time (*P* = 0.0006). Log-rank test was used to get the *P* value.

Among 140 PTB patients, the median follow-up time was 378 days (range: 1–468 days), and 13 deaths occurred (12 SE and 1 non-SE) as of August 30, 2023. Trend analysis showed a progressive increase in sPD-L1 levels from the HCs (*n* = 79), through Non-SE (*n* = 80), SE (*n* = 60), and finally to the dead group (*n* = 13) (Figure 5A, *p* < 0.0001). PTB patients were subdivided into high sPD-L1 and low sPD-L1 groups based on the Youden’s index-drive cut off value (445.1 pg/mL). Survival analysis revealed significantly shorter survival times in groups with higher sPD-L1 (Figure 5B, *p* = 0.0006).

## Discussion

Tuberculosis (TB) is an infectious disease caused by bacillus Mtb, before the pandemic of COVID-19, TB remains the leading cause of death worldwide from a single infectious pathogen. Although the disease can be cured with appropriate medication, it is a serious infectious disease worldwide, with an estimated 10.6 million people were diagnosed with TB, and 1.6 million people died from TB in 2022 [18, WHO report 2024]. The occurrence and development of tuberculosis is largely dependent on host immune status. Most people could eliminate Mtb and control the infections, manifesting as asymptomatic or mild symptoms, about 3% develop to severe TB [17]. Severe cases exhibit irreversible lung structure damage and functional impairment, significantly compromising prognosis and quality of life [10]. Therefore, identifying biomarkers to stratify disease severity and predict outcomes is thus pivotal for optimizing therapeutic strategies [19–21].

Nowadays, it is known that immune checkpoints regulate inflammation through costimulatory and coinhibitory signals [22,23]. Costimulatory molecules are necessary for T cell proliferation, activation, and polarization, whereas coinhibitory molecules, usually mediate interactions between antigen-presenting cells (APCs) and T cells and plays a vital role in maintaining immune homeostasis [22–24]. It has been reported that sPD-L1 arises *via* metalloprotease-mediated shedding, such as metalloproteinases (MMP) and metalloproteases (ADAM), or alternative splicing [3,25]. But the structural heterogeneity of sPD-L1, and associated functional plurality, should be considered when considering sPD-L1 as a biomarker[26–28]. Blood sPD-L1 could be detected using commercial kit or PD-L1 antibodies, but neither of them indicates the characterization of sPD-L1detected. Different kits and mAbs might draw distinct results from the same group of patients [27]. In this study, we used both fluorescent microspheres and previously developed sandwich ELISA to detect sPD-L1 levels in PTB patients. The sandwich ELISA had similar sensitivity and specificity to commercial kit, especially our mAbs could detect and quantify the functional sPD-L1 [12].

Elevated sPD-L1 levels have been documented in more than 20 diseases, including cancers [3,8–11], inflammatory diseases [12,14,16], autoimmune disorders [29,30], viral infections [31–33], and sepsis [13,34], where they often correlate with advanced disease and poor prognosis [10–13,34]. In sepsis, serum sPD-L1 elevation may reflect disease severity and serve as an independent prognostic marker [13]. In acute pancreatitis, serum sPD-L1 was significantly increased, especially in those with infectious complications, and negatively correlated with lymphocyte count. sPD-L1 appears to be involved in the development of immunosuppression in acute pancreatitis [14]. In this study, we found that sPD-L1 were significantly increased in PTB patients than those in HCs, especially in SE patients. Multivariable analysis showed that sPD-L1 was an independent predictor of severe PTB. In addition, PTB patients with higher levels of sPD-L1 had shorter survival time. Elevated sPD-L1 correlated with severe disease and poor prognosis in PTB in line with other disease. Furthermore, in PTB patients, plasma sPD-L1 levels positively correlated with inflammatory markers, such as hs-CRP, NEU%, NLR, ADA, and LDH. High levels of sPD-L1 may indicate of PTB patients’ systemic inflammatory state. Perhaps similar to acute pancreatitis, elevated sPD-L1 levels in PTB may involve in the immunosuppression and unsolved inflammation, facilitating Mtb persistence and tissue damage. sPD-L1 was an potential biomarker for prediction of severity and poor prognosis of PTB.

Pleural effusion (PE), a common clinical manifestation of diverse etiologies, most frequently results from TB or lung cancer. Traditional TPE diagnosis mainly relies on effusion acid fast staining, Mtb culture, and nucleic acid amplification test, but remains challenging due to low sensitivity (TPE was paucibacillary) and delayed culture results [35–37]. However, biomarkers in pleural fluid or blood have the advantages of easy accessibility and low invasiveness, diagnosis of TPE or MPE could be made on basis of biochemical criteria, such as ADA [35]. In this study, we found that the sPD-L1 levels in TPE was significantly higher than those in MPE, with diagnostic accuracy similar with ADA (AUC of 0.9837 versus 0.9859). These indicate sPD-L1 might be a useful marker for differentiating TPE from MPE in clinical practice.

At present, immunotherapy is gaining increasing attention in tuberculosis. The comprehensive treatment combining chemotherapy with adjuvant immunotherapy is advocated. Especially for multidrug-resistant and extensively drug-resistant tuberculosis, the demand for immunotherapy is most evident [38]. For PTB patients with immune dysfunction, such as patients with diabetes or HIV infection, reasonable use of immune agents can improve their prognosis[39]. Although sPD-L1 contributes to T cell exhaustion, the blocking of PD-1/PD-L1 in TB is impractical, because unleashing the restriction of this checkpoint exacerbates disease progression [40,41]. In this study, sPD-L1 is employed as a biomarker of immune dysregulation characterized by non-specific inflammation and impaired adaptive immunity, rather than as a therapeutic target, underscoring its potential to inform risk stratification or provide signal timely for high-risk patients to carry out adjuvant therapy.

There are some limitations in our study. First, our study involved a single center and further multicenter clinical studies with larger cohorts are required to validate our results. Second, further research is required to explore roles of sPD-L1 in the pathogenesis of PTB.

In conclusion, plasma sPD-L1 may be a predictive marker for disease severity and poor outcomes in PTB patients. Pleural effusion sPD-L1 levels might be an adjunctive marker for differentiating TPE from MPE.

## Supplementary Material

Supplementary_Table_1.docx

## Data Availability

The data that support the findings of this study are available from the corresponding author upon reasonable request.
