# Supplementary material for: Soluble programmed cell death ligand-1 as a predictive biomarker for severity and poor prognosis in pulmonary tuberculosis
Source: Ann Med. 2025 Jul 9;57(1):2527364. doi: 10.1080/07853890.2025.2527364 (PMC12247086; doi:10.1080/07853890.2025.2527364)
Supplement: Supplementary_Table_1.docx [file IANN_A_2527364_SM8276.docx]

Supplementary Table 1. Analysis of the correlation between clinical symptoms and five factors in PTB patients

|  | Plasma Cohort | |  | Correlation with sPD-L1 |
| --- | --- | --- | --- | --- |
|  | non-SE（n=80） | SE（n=60） | P value |  |
| Gender |  |  |  |  |
| Male/Female | 51/29 | 41/19 | 0.575 | P=0.830 |
| Age | 54.15±20.35 | 55.56±19.08 | 0.395 | P=0.414 |
| Signs and symptoms |  |  |  |  |
| Fever（%） | 21.25% | 43.33% | 0.006 | P=0.472 |
| Chest tightness（%） | 21.25% | 20.00% | 0.858 | P=0.832 |
| Cough（%） | 67.50% | 60.00% | 0.363 | P=0.108 |
| Gasp（%） | 6.25% | 23.33% | 0.007 | P=0.764 |
| TB inspection items |  |  |  |  |
| Smear（%） | 42.50% | 60.00% | 0.018 | P=0.321 |
| Culture（%） | 60.00% | 68.33% | 0.314 | P=0.427 |
| Xpert（%） | 78.75% | 81.67% | 0.672 | P=0.122 |
| Drug resistance（%） | 21.25% | 26.67% | 0.459 | P=0.657 |

Results are showed as number (%) or mean ± standard deviation(SD).
